# Effects of non-invasive vagus nerve stimulation on pupil dilation are dependent on sensory matching

**DOI:** 10.1016/j.isci.2026.114795

**Published:** 2026-01-24

**Authors:** Cecilia Vezzani, Rae-Marie Breakspear, Lilly Thurn, Ulrich Ettinger, Anne Kühnel, Nils B. Kroemer

**Affiliations:** 1 Section of Medical Psychology, Department of Psychiatry and Psychotherapy, University of Bonn, University Hospital Bonn, 53127 Bonn, North Rhine-Westfalia, Germany; 2 Department of Psychiatry and Psychotherapy, Tübingen Center for Mental Health, University of Tübingen, 72076 Tübingen, Baden-Württemberg, Germany; 3 German Center for Mental Health (DZPG), 72076 partner site Tübingen, Baden-Württemberg, Germany; 4 German Center for Diabetes Research (DZD), 85764 Neuherberg, Bavaria, Germany; 5 Department of Psychology, University of Bonn, 53111 Bonn, North Rhine-Westfalia, Germany

**Keywords:** Neurology, eye observable entity, affect, procedure by device, sensory neuroscience

## Abstract

Transcutaneous auricular vagus nerve stimulation (taVNS) is a promising tool to modulate motivation and affect, with prior studies showing greater pupil dilation compared to sham. However, sensory differences between conditions may confound results. In a randomized crossover study with 94 participants, we applied right-sided pulsed taVNS (1 s, 20 Hz, and 400 μs) and calibrated stimulation amplitude to match subjective sensation. Contrary to expectations, taVNS did not significantly increase pupil dilation versus sham. However, when participants perceived sham as less intense, taVNS effects were stronger. Differences in perceived sensation between taVNS and sham were mainly linked to sham-induced pupil dilation. Our findings suggest that right-sided pulsed taVNS only leads to greater pupil dilation when a sensory mismatch occurs, underscoring the difficulty of creating an effective sham.

## Highlights

• taVNS does not increase pupil dilation vs. sham when sensation is matched
• Differences in sensation for taVNS vs. sham are linked to sham-induced pupil dilation
• Right-ear taVNS reduced left-right pupil correlation after stimulation
• Sensory matching is critical for using pupil dilation as a taVNS biomarker

## Graphical abstract

**Figure:**
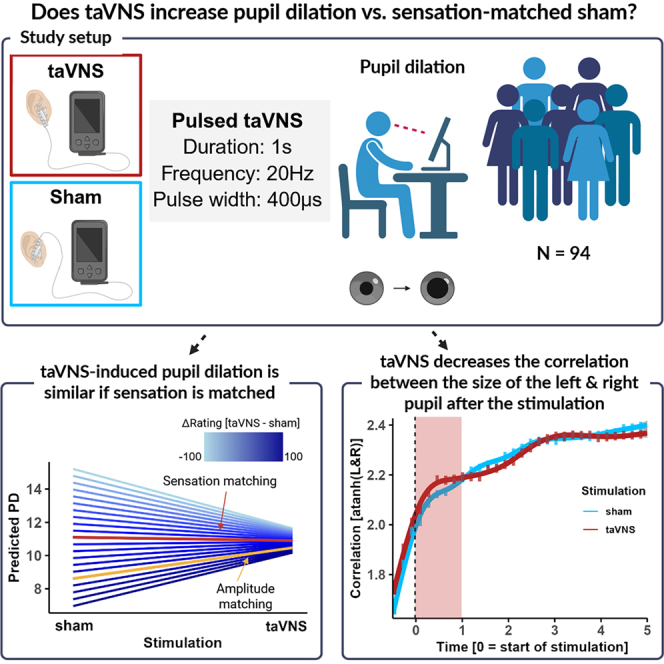

## Introduction

The vagus nerve plays a fundamental role in the communication between the body and the brain via the nucleus of the solitary tract (NTS).^1^ Bodily signals support homeostatic and allostatic processes, leading to neuromodulatory effects on motivation, cognition, and affect.^2^^,^^3^^,^^4^^,^^5^^,^^6^^,^^7^ To non-invasively activate vagal afferent projections to the brainstem, transcutaneous auricular vagus nerve stimulation (taVNS) has emerged as a promising technique. By applying electrical stimulation to the auricular branch of the vagus nerve, it has been repeatedly shown that brain responses in the NTS and a larger vagal afferent network can be altered.^8^^,^^9^^,^^10^^,^^11^ However, not every participant shows the expected marked activation of the primary target in the brain,^11^^,^^12^ raising questions about inter-individual variability that may explain mixed effects in studies focusing on clinical or behavioral outcomes. Consequently, the identification of a scalable biomarker for the successful activation of the vagus nerve in individuals would facilitate the future evaluation of interventions.^13^

One easily accessible biomarker is taVNS-induced pupil dilation.^13^^,^^14^^,^^15^ Vagal afferents project to the locus coeruleus (LC) via the NTS,^16^^,^^17^^,^^18^ and taVNS activates the LC.^18^ Pupil dilation reflects noradrenergic activity in the LC^19^ and cholinergic activity in the forebrain,^20^^,^^21^ making it a promising indirect biomarker for vagal activity. Preclinical work in rodents using invasive vagus nerve stimulation (VNS) has shown phasic firing in the LC^22^ and graded pupil dilation in response to increasing stimulation amplitude.^20^^,^^23^ In humans, invasive VNS also elicits pupil dilation,^24^^,^^25^^,^^26^ although one study found no VNS-induced effect.^27^ However, studies using non-invasive VNS in humans have yielded mixed results, partly due to different taVNS protocols.^13^^,^^28^ Studies using a conventional protocol (i.e., biphasic stimulation with 25 Hz and 250 μs pulse width for 30 s ON/30 s OFF^29^^,^^30^^,^^31^^,^^32^^,^^33^) have reported inconclusive results, likely because taVNS does not affect tonic pupil responses.^31^^,^^34^^,^^35^ Instead, studies using a pulsed protocol (i.e., shorter pulses ranging between 0.5 and 5 s delivered at regular or irregular intervals^14^^,^^15^^,^^34^^,^^36^^,^^37^^,^^38^ have been more effective in eliciting phasic pupil dilation,^14^^,^^15^^,^^33^ leading to significant effects in a recent meta-analysis.^28^

Despite these promising findings, several open questions remain. First, the largest study using pulsed taVNS included 58 participants, while many had fewer than 30, and were not sufficiently powered given the estimated effect size.^28^ Second, all studies applied taVNS at the left ear, even though studies suggest the lateralization of taVNS effects.^5^^,^^6^^,^^11^^,^^39^^,^^40^ Third, unlike invasive VNS, taVNS activates sensory nerves. This produces a pricking sensation that triggers a physiological orienting response and pupil dilation.^41^^,^^42^ The commonly selected sham stimulation at the earlobe also induces this orienting response,^15^^,^^43^ as the earlobe is innervated by the great auricular nerve, which also transmits sensory information.^41^^,^^42^^,^^44^ However, many studies have used the same stimulation amplitude for both conditions without accounting for differences in sensation across conditions,^30^^,^^31^^,^^33^^,^^35^^,^^37^^,^^38^^,^^45^ in contrast to best-practice guidelines.^46^ Previous studies have shown that differences in sensation accounted for taVNS-induced increases in pupil dilation,^37^ and even initial sensation matching between taVNS and sham conditions may not be sufficient.^32^ To summarize, despite the promising meta-analytic evidence in favor of pulsed taVNS, many questions regarding the robustness in larger samples, generalizability to right-sided stimulation, and confounding due to sensory aspects remain.

**Figure 1:**
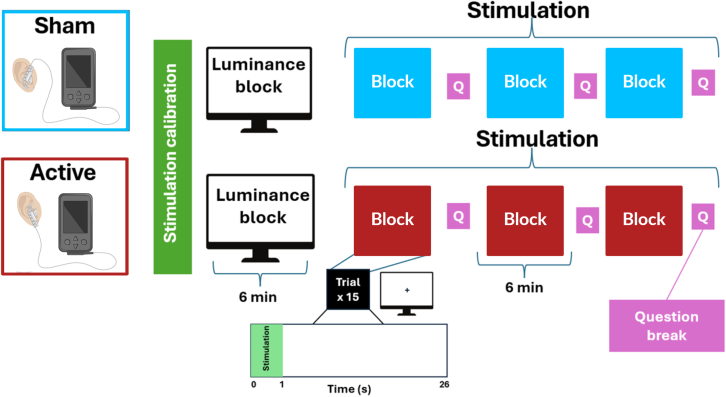
Overview of the study design and stimulation conditions. After placing the electrode, participants completed a stimulation calibration to determine the optimal stimulation amplitude for the following blocks. The stimulation calibration was followed by an initial luminance block (15 trials of 15 s) without stimulation. After the luminance block, participants underwent three stimulation blocks of approximately 6 min each, during which they received short taVNS pulses of 1 s with an interstimulus interval of 25 s. They received 15 stimulations in each block, and the block was followed by a sensation rating. After the three blocks and a short break, the electrode was placed for the other condition (sham or taVNS), and the same procedure was repeated. Figure adapted from Biorender.

To address these gaps, we tested the effects of pulsed taVNS (1 s, 20 Hz, 400 μs, individually calibrated amplitude applied at the right ear) on pupil dilation in a large sample of 94 healthy participants. To determine if taVNS-induced changes depend on individual variability in sensation matching, participants repeatedly rated the sensation of the stimulation over time (Figure 1). We hypothesized that pulsed taVNS compared to sham would lead to higher pupil dilation. Moreover, we expected that the rated sensation of the stimulation would modulate the difference between taVNS and sham on pupil dilation. We found that taVNS did not elicit higher pupil dilation than sham if differences in sensation ratings were accounted for. However, when stimulation amplitude was statistically matched between taVNS and sham (i.e., the same stimulation amplitude; differences in sensation), the predicted marginal means showed a significant taVNS-induced response vs. sham, consistent with previous amplitude-matching studies. Since taVNS-induced differences in pupil dilation were mostly driven by a larger variability in sham responses, our study emphasizes the importance of optimizing the sham condition for pulsed taVNS protocols.

## Results

### Transcutaneous auricular vagus nerve stimulation does not induce increased pupil dilation

**Figure 2:**
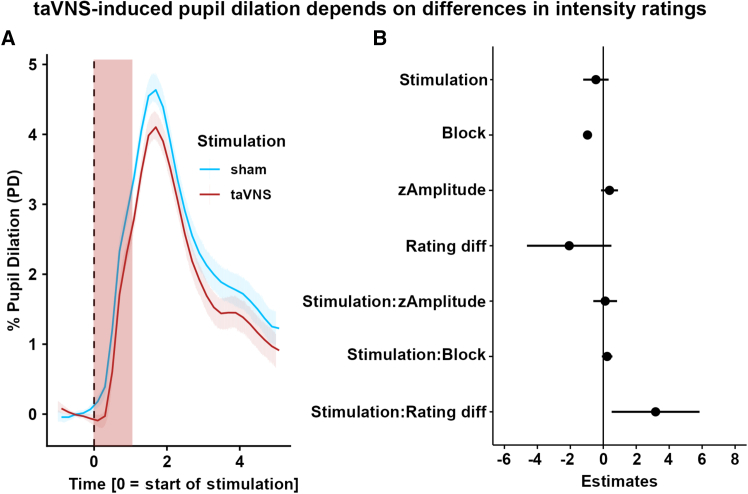
taVNS does not induce larger pupil dilation compared to sham (A) Changes in pupil dilation for taVNS (red line) vs. sham (blue line) over time, showing no significant difference in pupil dilation between conditions. Pupil dilation is expressed in percentage change over baseline. Highlighted in red is the stimulation pulse. (B) Mixed-effects model for the maximum pupil dilation, showing a negative effect of block, *b*(89.5) = −0.97, 95% CI [-1.23; −0.71], *p* < 0.001 (Figure S2A), and a positive interaction effect of stimulation and rating differences (Rating diff), *b*(95) = 3.39, 95% CI [0.62; 6.17], *p* = 0.019. Error bars show 95% confidence intervals for the estimates.

To test the effects of taVNS, we analyzed pupil dilation within the 5 s post-stimulation time window using mixed-effects models. Contrary to our hypothesis, taVNS did not elicit greater pupil dilation compared to sham during the post-stimulation period, *b*(235.3) = −0.42, 95% CI [-1.30; 0.46], *p* = 0.35 (Figures 2A and 2B). To account for individual differences in sensation ratings between conditions, we included the average differences in sensation ratings between taVNS and sham. We found that the effects of taVNS on pupil dilation were larger for participants with a larger difference in ratings (reflecting average differences in sensation across the three blocks between taVNS and sham), Stimulation × Rating diff *b*(95) = 3.39, 95% CI [0.62; 6.17], *p* = 0.019, suggesting that sensation ratings partly explain taVNS-induced pupil dilation. Since trial-wise maximum pupil dilation might miss effects related to a prolonged pupil dilation, we repeated the analysis with area under the curve as the outcome. Again, we found no significant effect of taVNS, *b*(268.7) = −2.68, 95% CI [-5.95; 0.58], *p* = 0.11 (Figure S1A, see https://osf.io/7h6f4 for model output). To exclude that carry-over effects on baseline pupil dilation alter the contrast between conditions, we also analyzed trial-wise baseline values and found no differences between sham and taVNS, *b*(93) = 3.03, 95% CI [-61.54; 67.61], *p* = 0.93 (Figure S1B).

**Figure 3:**
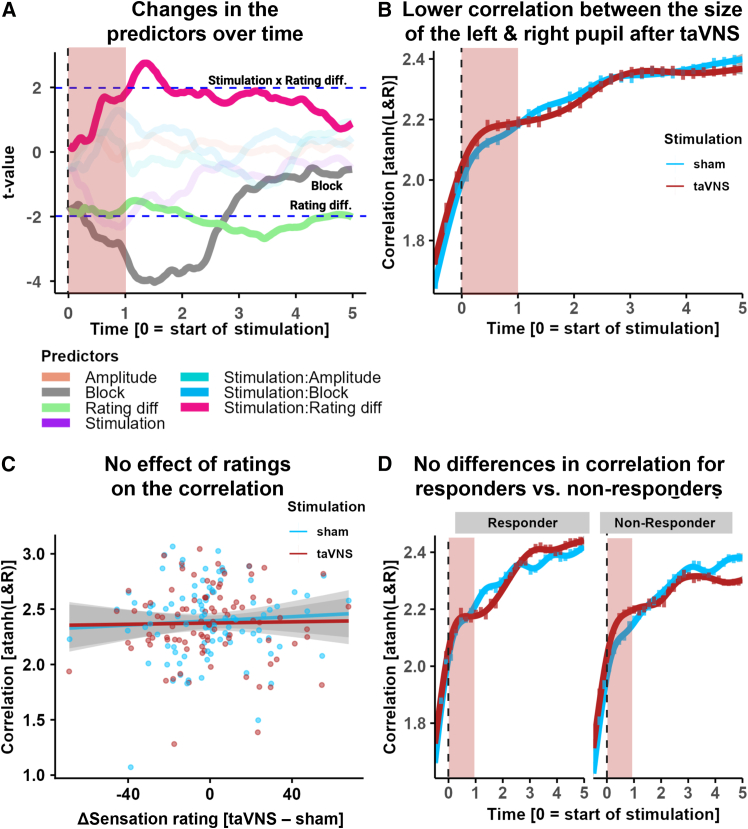
Time series analysis and correlation of the size of the left and right pupil (A) Time-resolved analyses of taVNS-induced changes in pupil dilation show an early modulation by rating differences (Rating diff.). Additional effects that cross the significance threshold are highlighted. The red overlay shows the time of stimulation. Data were analyzed in bins of 100 ms. Dashed blue lines indicate the two-tailed critical t-value corresponding to α = 0.05. (B) Lower correlation for taVNS vs. sham between the dilation of the left and right eye after the stimulation pulse, Stimulation × Time *b*(93) = −0.06, 95% CI [-0.10; −0.02], *p* = 0.004). Correlation values were atanh-transformed for parametric analyses. In red, the stimulation time is highlighted. Error bars reflect bootstrapped 95% confidence intervals. (C) No effect of differences in perceived sensation on the correlation between pupil dilation after stimulation (Rating diff × Time *b*(92) = 0.02, 95% CI [-0.15; 0.19], *p* = 0.83; Rating diff × Time × Stimulation *b*(92) = −0.06, 95% CI [-0.24; 0.11], *p* = 0.47. (D) The difference in the correlation of left and right pupil size between taVNS and sham does not vary significantly between responders and non-responders over time (Stimulation × Time *b*(92) = 0.07, 95% CI [-0.01; 0.16], *p* = 0.069). Error bars reflect bootstrapped 95% confidence intervals.

To explore whether our aggregate measures missed effects at specific times during the pupil response, we conducted time series analyses estimating separate mixed-effects models for pupil dilation binned into 100 ms intervals. Mirroring the main results, there was no significant effect of taVNS at any time point (*p*s > 0.05; Figure 3A). The interaction between stimulation and sensation rating differences was most pronounced after the stimulation pulse (significant interaction between 1.0 s and 1.6 s; FDR-corrected *p*s < 0.05, peak at 1.3 s: *b*(94.5) = 7.84, 95% CI [2.39; 13.29], FDR-corrected *p* = 0.005; Figures 3A and S2B).

### Transcutaneous auricular vagus nerve stimulation reduces the correlation between pupil dilation traces of the left and right eye

To investigate the lateralized effects of taVNS on pupil dilation, we examined the correlation between pupil responses of the left and right eye. Since we only stimulated at the right ear, any lateralized effect of the stimulation would briefly reduce the correlation of the two pupil dilation traces. In line with lateralized effects, we found that pulsed taVNS reduced the correlation (atanh-transformed for parametric statistics) between pupil dilation responses after the stimulation period (Stimulation × Time *b*(93) = −0.06, 95% CI [-0.10; −0.02], *p* = 0.004; Figure 3B) when correlations overall increased compared to the acute stimulation phase (Time *b(*93) = 0.34, 95% CI [0.30; 0.38], *p* < 0.001; Figure 3B). This difference in the correlation of the left and right eye between taVNS and sham did not significantly vary between responders (i.e., individuals with greater maximum pupil dilation for taVNS vs. sham in the 5 s post stimulation phase) and non-responders (Stimulation × Time × Responders *b*(92) = 0.07, 95% CI [-0.01; 0.16], *p* = 0.069; Figure 3D). We also tested whether differences in perceived sensation between conditions affected the correlation between pupil dilations after stimulation, but this was not the case (Rating diff × Time *b*(92) = 0.02, 95% CI [-0.15; 0.19], *p* = 0.83; Rating diff × Time × Stimulation *b*(92) = −0.06, 95% CI [-0.24; 0.11], *p* = 0.47; Figure 3C).

### Differences in sensation contribute to transcutaneous auricular vagus nerve stimulation-induced pupil dilation

**Figure 4:**
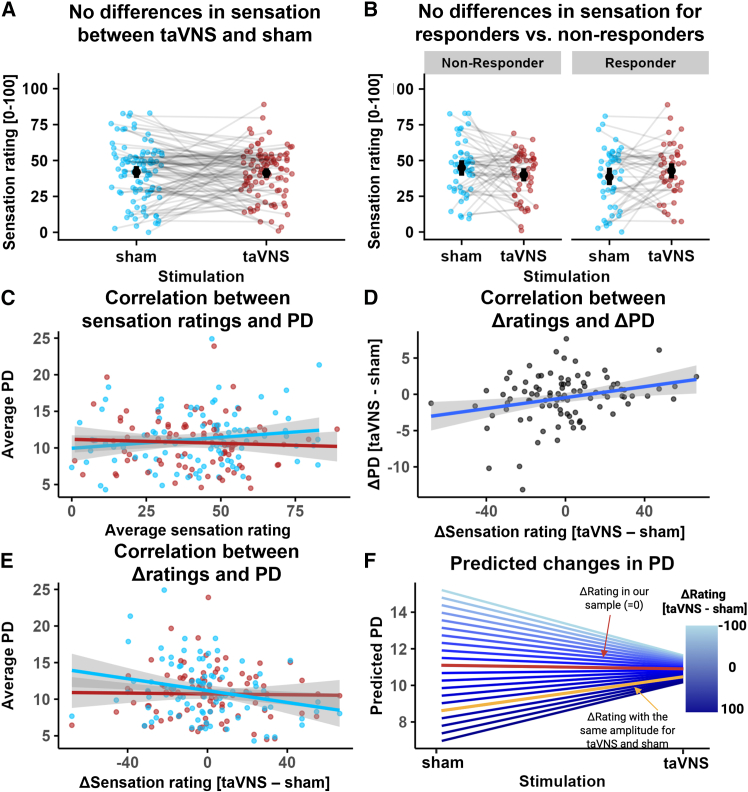
Differences in sensation explain taVNS-induced pupil dilation (PD) (A) No differences in sensation for taVNS (red) and sham, *b*(92) = −0.55, 95% CI [-5.37; 4.27], *p* = 0.82. Error bars depict 95% bootstrapped CIs. (B) No significant differences in sensation between responders and non-responders, *b*(92) = −9.31, 95% CI [-18.84; −0.21], *p* = 0.055). Error bars depict 95% bootstrapped CIs. (C) No significant correlation between sensation and PD for taVNS, *r*(92) = −0.06, 95% CI [-0.26; 0.15], *p* = 0.60, and sham, *r*(92) = 0.16, 95% CI [-0.05; 0.35], *p* = 0.13. (D) Positive correlation, *r*(92) = 0.27, 95% CI [0.08; 0.45], *p* = 0.008) between differences in sensation (taVNS – sham; Δrating) and differences in average PD. (E) Negative correlation for sham, *r*(92) = −0.25, 95% CI [-0.43; −0.05], *p* = 0.013, but not taVNS (red; *r*(92) = −0.02, 95% CI [-0.22; 0.18], *p* = 0.85) between differences in sensation (Δrating) and PD. (F) Prediction of changes in PD for sham and taVNS based on differences in sensation (Δrating), calculated by estimating marginal means. Red line: sensation differences in our sample (Δrating = −0.46); yellow line: sensation differences corresponding to an amplitude difference of 0 in our sample (Δrating = ∼60).

We first compared sensation ratings between taVNS and sham and observed no differences overall, *b*(92) = −0.55, 95% CI [-5.37; 4.27], *p* = 0.82 (Figure 4A), and no differences in changes over blocks (Figure S3A, see https://osf.io/7h6f4). Although on average the sensation ratings were comparable, interindividual differences in perceived sensation may still contribute to interindividual variability in the taVNS-induced pupil response. Next, we examined whether “responders” (i.e., greater maximum dilation for taVNS vs. sham in the 5 s post stimulation, *n* = 44) and “non-responders” (*n* = 50) showed differences in sensation ratings. The difference was in the expected direction (i.e., responders reported slightly higher sensation ratings for taVNS vs. sham), but it did not reach significance, *b*(92) = −9.31, 95% CI [-18.84; 0.21], *p* = 0.055 (Figure 4B). However, this does not provide evidence for the null hypothesis, as Bayesian analysis showed anecdotal evidence for a difference in sensation ratings, BF_10_ = 1.07. Neither the taVNS- or sham-induced pupil dilation was correlated with the corresponding sensation, *r*_*taVNS*_(92) = −0.06, 95% CI [-0.26; 0.15], *p* = 0.60; *r*_*sham*_(92) = 0.16, 95% CI [-0.05; 0.35], *p* = 0.13 (Figure 4C), and the two correlations did not differ, *Steiger’s Z* = −1.45, *p* = 0.15. However, there was a negative correlation between differences in sensation ratings (taVNS – sham) and pupil dilation in the sham condition only, *r*_*sham*_(92) = −0.25, 95% CI [-0.43; −0.05], *p* = 0.013; *r*_*taVNS*_(92) = −0.02, 95% CI [-0.22; 0.18], *p* = 0.85, (Figure 4E), as well as a positive correlation between differences in sensation ratings and differences in pupil dilation (taVNS – sham), *r*(92) = 0.27, 95% CI [0.08; 0.45], *p* = 0.008 (Figure 4D), reflecting the significant Stimulation × Rating diff interaction in the main model (Figure 2B).

To compare our results with previous studies using different matching procedures, we estimated marginal means of the fitted linear mixed-effects model to predict average pupil dilation based on sensation differences between taVNS and sham (Figure 4F). If sensation is comparable on average across all task blocks and participants as in our sample (Δrating = −0.46; Figure S3A), taVNS-induced pupil dilation does not differ from sham (Figure 2). However, without sensation matching at the beginning of the experimental session (using the same stimulation amplitude for taVNS and sham instead), this would lead to a larger pupil dilation for taVNS compared to sham, as predicted by estimated marginal means (*b*_*taVNS-sham*_ = 1.86, *p* = 0.037; yellow line in Figure 4F). This is in line with findings from three other studies.^31^^,^^37^^,^^38^ Crucially, taVNS-induced pupil dilation was highly similar across all sensation ratings, whereas sham-induced pupil dilation was strongly dependent on differences in sensation. In other words, sham stimulation might produce more variable pupil dilation responses depending on the sensation-matching procedure compared to taVNS (Figure S4B).

### Control and sensitivity analyses

**Figure 5:**
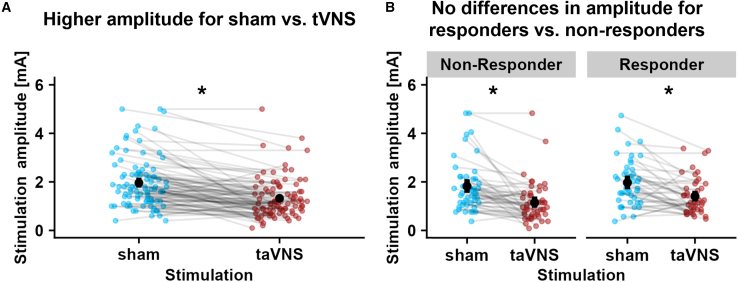
Differences in amplitude for taVNS vs. sham (A) Lower amplitude for taVNS (red) compared to sham (blue), *b*(92) = −0.64 mA, 95% CI [-0.82; −0.46], *p* < 0.001. (B) No differences in amplitude for taVNS vs. sham between responders and non-responders, *b*(92) = 0.12 mA, 95% CI [-0.25; 0.48], *p* = 0.53. Error bars depict 95% bootstrapped CIs. In all panels, significance levels are set at α < 0.05 and are calculated using linear mixed models (*lmerTest*) and the Satterthwaite method to determine the degrees of freedom.

To ensure that our results cannot be explained by confounds, we performed additional control analyses (see Figures S3B, S4, and S5 for sensitivity analyses relative to potential order effects and testing time). As expected,^14^^,^^15^^,^^29^^,^^32^ participants received a significantly lower stimulation amplitude during taVNS compared to sham, *b*(92) = −0.64 mA, 95% CI [-0.82; −0.46], *p* < 0.001 (Figure 5A; Table 1). Nevertheless, responders and non-responders did not differ in stimulation amplitude between sham and taVNS, Stimulation × Responder: *b*(92) = 0.12 mA, 95% CI [-0.25; 0.48], *p* = 0.53 (Figure 5B). taVNS-induced changes were also not meaningfully affected by the starting time of the session, age, and sex of the participants, as responders and non-responders did not differ in those characteristics (Table 1).

**Table 1:** Information regarding participants’ demographics and testing parameters, separated by responders and non-responders

	Responders	Non-responders	Test	*p*
*N*	44	50		
Female/Male Ratio	21/23	27/23	*χ*^*2*^ = 0.38	0.54
taVNS 1^st^ session (order)	24	22	*χ*^*2*^ = 1.04	0.31
Age	23.5 ± 3.19	24.7 ± 3.56	*t* = 1.35	0.18
Starting time	1:10 p.m. ± 3 h	12:47p.m. ± 2.30 h	*t* = 0.07	0.94
Stimulation amplitude (taVNS)	1.5 ± 0.8 mA	1.2 ± 0.9 mA	*t* = −1.41	0.16
Stimulation amplitude (sham)	2.0 ± 1.0 mA	1.9 ± 1.1 mA	*t* = −0.80	0.42
Stimulation sensation rating (taVNS)	42 ± 18	40 ± 16	*t* = −0.79	0.43
Stimulation sensation rating (sham)	38 ± 21	45 ± 19	*t* = 1.56	0.12

Data are reported as mean ± SD. Chi-squared tests were used to compare the distribution of participants. Independent t-tests were used for differences in age and starting times. t-values for stimulation amplitude and sensation were calculated with a mixed-effects model.

Moreover, we ran additional sensitivity analyses, including block-wise sensation ratings in our mixed-effects model instead of the average sensation rating differences. When fixed and random slopes for sensation ratings were included, higher sensation ratings were associated with larger pupil dilation across conditions, *b*(45.45) = 1.82, 95% CI [0.34; 3.31], *p* = 0.016. However, the interaction between stimulation and sensation ratings did not reach significance (*b*(483) = −2.29, 95% CI [-4.60; 0.03], *p* = 0.053; Figure S6), showing that higher sensation ratings were associated with greater pupil dilation, although this effect was somewhat weaker for taVNS. Although the interaction did not reach significance, the pattern reflects the observed association of pupil dilation in the sham condition with the rating differences in the main model and the corresponding estimated means. We further compared the main model (including rating differences) with the block-wise ratings model, finding no significant improvement in model fit (Rating differences model: AIC = 123,370, BIC = 123,486.2; block-wise rating model: AIC = 123,375, BIC = 123,522.2). Finally, to evaluate the robustness of our findings relative to the correlation between left and right pupil and ensure that including a brief baseline in the “early” time window (i.e., time bins from 1 s before stimulation onset until stimulation end) did not bias our outcome (Figures 3B and 3D), we compared our model with a temporally restricted model only including time bins from the start of stimulation. In line with the results reported in Figure 3B, we found that pulsed taVNS reduced the correlation between pupil dilation responses in the post stimulation period (Stimulation × Time *b*(93) = −0.05, 95% CI [-0.10; −0.01], *p* = 0.01), while across conditions, correlations increased after the stimulation phase (Time *b(*93) = 0.21, 95% CI [0.17; 0.25], *p* < 0.001).

## Discussion

Pupil dilation has been proposed as a promising biomarker for taVNS, particularly if shorter pulses are administered^32^^,^^37^^,^^47^ (see Pervaz et al.^28^ for an overview). Here, we used 1 s pulses at a frequency of 20 Hz to compare the effects of taVNS against sham on pupil dilation while matching the sensation ratings between conditions. Contrary to our hypothesis, taVNS did not elicit greater pupil dilation compared to sham, unless participants perceived the active stimulation as more intense on average throughout the session, despite initial sensation matching. Once we used sensation ratings to model observed differences in pupil dilation, our results recapitulate earlier findings with matched amplitude.^37^^,^^45^ Taken together, our study demonstrates the pivotal role of sensory aspects of both taVNS and sham for stimulation-induced pupil dilation, necessitating further improvements in design to provide a meaningful biomarker.

Invasive VNS reliably elicits pupil dilation in humans and animals, likely via noradrenergic LC signaling, without involving sensory pathways.^20^^,^^23^^,^^24^^,^^25^^,^^26^ However, the electrode placement in taVNS stimulation leads to a perceivable sensation accompanying both active and sham stimulation. To better dissociate the effects of stimulation amplitude and sensation ratings, we individually calibrated stimulation amplitudes while monitoring potential changes in sensation ratings throughout the session. As recently summarized by Pervaz et al.,^28^ 6 out of 10 pulsed taVNS studies used sensation matching. Unlike prior studies that used preset stimulation amplitude across participants (i.e., the same amplitude administered to all participants) for both conditions or only calibrated the amplitude of one of the conditions,^30^^,^^31^^,^^33^^,^^35^^,^^37^^,^^38^^,^^45^ we followed the procedure established for conventional taVNS and individually calibrated both taVNS and sham stimulation to match the sensation between conditions.^46^ Then, analogous to D’Agostini et al.^36^ and Ludwig et al.,^37^ we also collected sensation ratings at the end of each block. In line with Ludwig and colleagues,^37^ we observed that differences in taVNS-induced pupil dilation were correlated with changes in sensation ratings between conditions. The earlobe, which is often used for sham stimulation as it is not innervated by the vagus nerve, usually requires a higher amplitude compared to the cymba conchae to elicit comparable sensation ratings.^15^^,^^36^ Hence, an imbalance in sensation ratings between conditions could inflate taVNS-induced pupil dilation.^36^^,^^37^ Perhaps surprisingly, our study shows that the inter-individual variability in taVNS-induced pupil dilation is largely driven by changes in sensation ratings for sham. This raises questions about the potential variability of sham positions of the electrode, which is supported by related findings showing that earlobe stimulation exerts regulatory effects on physiological indices.^48^ In other words, sham stimulation at the earlobe should not be considered a neutral control, and while the stimulation of the great auricular nerve could be a useful control condition for fMRI or behavioral studies, it may not be ideal to develop a biomarker based on pupil dilation.^44^ Alternatively, differences in the correlation of pupil dilation traces of the left and the right eye could be useful to identify effective stimulation of one side of the vagus, potentially providing an index that is less confounded by differences in sensation ratings. To conclude, taVNS-induced pupil dilation appears to be largely driven by the contrast to sham, which is highly dependent on proper sensation matching.

To conclude, pupil dilation is a candidate biomarker for taVNS, and a recent meta-analysis suggested that pulsed taVNS induces pupil dilation across studies. However, our study shows that taVNS-induced pupil dilation only exceeded sham if differences in sensation ratings arose despite the initial sensation-matching procedure. Consequently, sensory aspects of the stimulation play a fundamental role in determining the differences in pupil dilation between taVNS and sham, which both stimulate sensory nerves. We found that sham-induced pupil dilation was highly variable depending on the rated sensation differences, suggesting that differences in the positioning of the electrode for sham stimulation could contribute to inter-individual differences. Additionally, taVNS-induced differences between the pupil dilation traces of the left and the right eye argue for a lateralized effect independent of differences in sensation ratings that may have the potential to identify the effective stimulation of vagal afferents. Future research should improve protocols to ensure a stable sensation, and potential variability due to the sham position of the electrode requires follow-up work to identify whether there are unintended indirect effects on vagal afferents due to the stimulation of the great auricular nerve.^44^

### Limitations of the study

Despite the strengths of our study, such as a large sample and a within-subject manipulation, several limitations need to be considered. First, our chosen parameters for pulsed taVNS were based on a small pilot, where we applied different combinations of frequency and duration and found that most participants responded to the 1 s/20 Hz combination. It is possible that different pulse settings would increase the number of responders. Second, although the order effects were small and mostly non-significant, they tended to have an attenuating effect on taVNS-induced pupil dilation. Hence, running sessions on different days or with a longer washout period on the same day may lead to larger differences. Likewise, we also observed decreases in pupil dilation over blocks, pointing to potential habituation or desensitization over time. Third, the standardized calibration procedure was previously established for conventional taVNS, but the observed changes in sensation ratings over time suggest that it does not sufficiently account for the inter-individual variability for pulsed taVNS. Given the effect of sensation differences on pupil dilation, future studies should focus on improving protocols that ensure a stable and comparable sensation between conditions (i.e., participants in crossover designs), within conditions (i.e., between participants, e.g., due to variability in the exact positioning of the electrode), and over time (i.e., variability in habituation). Our results suggest that intraindividual changes in perceived sensation across blocks were relatively minor and are unlikely to explain the observed effects. Instead, interindividual differences (potentially related to electrode placement) may have contributed more strongly to variability in perceived sensation. In the sham condition, small differences in positioning on the earlobe may alter the local stimulation of adjacent nerve branches or cause slight electrode movements during the task, thereby affecting the perceived intensity. To minimize such variability, we recommend careful standardization of electrode placement, especially for sham stimulation, and we suggest recalibrating the stimulation amplitude before each experimental block to maintain comparable sensations over time. Fourth, while our final calibration sensation (mild pricking sensation; average calibration amplitude for taVNS was 1.3 mA ± 0.9, for sham 2.0 mA ± 1.0) is in line with several previous studies applying conventional^4^^,^^12^^,^^29^^,^^32^ and pulsed^36^ taVNS using a comparable taVNS device, other studies investigating pupil dilation used a shorter pulse width and reported higher average amplitudes (on average ∼2.25 mA for taVNS and 2.9 mA for sham).^14^^,^^15^^,^^45^ Since longer pulse width corresponds to a greater intensity at the same amplitude, it is currently not known which setting is optimal to drive pupil dilation. Differences in stimulation protocols may partially explain the differences in effect sizes between studies. Finally, we used right-sided taVNS, in contrast to most studies that combine taVNS and eye-tracking.^28^ Since the right and left vagus nerves differentially innervate organs, and previous studies have shown lateralized effects,^5^^,^^49^ it remains to be determined if left and right taVNS elicit comparable pupil dilation responses.

## Resource availability

### Lead contact

Further information and requests for resources should be directed to Prof. Dr. Nils B. Kroemer (nkroemer@uni-bonn.de).

### Materials availability

The preprocessed data reported in this article are publicly available on OSF. The link is listed in the key resources table.

### Data and code availability

• Preprocessed data have been deposited in an OSF archive (https://osf.io/7h6f4) and are publicly available. The link is listed in the key resources table.
• All original codes have been deposited in an OSF archive (https://osf.io/7h6f4) and are publicly available. The link is listed in the key resources table.
• Any additional information required to reanalyze the data reported in this article can be made available by the lead contact upon request.

## Acknowledgments

The study was supported by the BONFOR Grant O-128.0101 and the German Research Foundation (10.13039/501100001659DFG), grant KR 4555/10-1.

## Author contributions

N.B.K. and A.K. were responsible for the study concept and design. C.V., L.T., and R.M.B. collected data under supervision by N.B.K. C.V., A.K., and N.B.K. conceived the methods, A.K. processed the data, and U.E. supported the development of the methods. C.V. performed the data analysis, and A.K. and N.B.K. contributed to the analyses. C.V., A.K., and N.B.K. wrote the article. All authors contributed to the interpretation of findings, provided critical revision of the article for important intellectual content, and approved the final version for publication.

## Declaration of interests

The authors declare no competing interests.

## Declaration of generative AI and AI-assisted technologies in the writing process

The authors acknowledge the use of ChatGPT and Grammarly for language editing. After using this tool, the authors reviewed and edited the content as needed and take full responsibility for the content of the publication.

## STAR★Methods

### Key resources table

**Table:** 

REAGENT or RESOURCE	SOURCE	IDENTIFIER
**Deposited data**		
Preprocessed data and code	OSF archive	https://osf.io/7h6f4

**Software and algorithms**		

MATLAB	The MathWorks	v. R2023b
EyeLink Software	SR Research, Kanata, ON, Canada	EyeLink 1000 eye-tracker
R Software	R Foundation for Statistical Computing, Kaysville, UT, USA	v. 4.5.1

**Other**		

tVNS technologies device	tVNS Technologies®, Reichenschwand, Germany	tVNS technologies R

### Experimental model and study participant details

We recruited 94 healthy participants (47 women, self-reported biological sex, *M*_Age_ = 24.3 years, ±3.4) via public announcement, through flyers handed out in Bonn, as well as through social media posts. To be eligible, they had to fulfill the following inclusion criteria: 1) between 18 and 35 years of age, 2) BMI between 18.5 and 30 kg/m^2^, 3) no history of neurological disorders, 4) no history of schizophrenia, bipolar disorder, or substance use disorder, 5) normal or corrected to normal vision, 6) no implants (e.g., cochlear implants, pacemakers, cerebral shunts). To take part, all participants had to provide written informed consent prior to the first session. The influence of sex on pupil dilation was analyzed as part of the results, and no significant differences were found (Table 1). No information on gender, race, or ancestry was collected from participants in accordance with local regulations. However, participants were either German or English speaking and were mostly students including international students from the University of Bonn. After the experimental session, participants were categorized into two groups (Responders, Non-responders) based on whether they reported greater maximum pupil dilation for taVNS vs. sham in the 5s post stimulation phase. The study protocol was approved by the ethics committee at the Medical Faculty of the University of Bonn (Reference code 492/22-EP), and it was preregistered on clinicaltrials.gov (ID NCT06205108).

### Method details

#### Experimental procedure

The study consisted of one session lasting about 1.5h, and participants received taVNS and sham stimulation in a randomized order. On the day of the session, participants were asked to abstain from drinking coffee and consuming nicotine-containing products for at least 2h before the start of the experiment. Once in the experiment room, participants were introduced to the study procedure and then provided written informed consent to participate. After taking anthropometric measures (i.e., height, weight, hip and waist circumference), participants answered the positive and negative affect schedule (PANAS^50^) about their current mood and questions regarding their metabolic state (e.g., hunger, satiety^12^^,^^50^^,^^51^^,^^52^) on a visual analog scale (VAS, 0–100).

Next, participants were asked to remove any earrings, and the taVNS device was placed at the participant’s right ear according to the condition: taVNS (cymba conchae) or sham (earlobe). Before placing the electrode, we cleaned the skin of the ear with an alcohol pad. To determine an individualized stimulation amplitude that activated vagal afferent projections, a calibration procedure was performed twice during the experimental session, once before the first condition and a second time after the first half of the experiment and before the start of the second condition. During the calibration, participants received increasing stimulation amplitudes (0.1 mA increments) for the duration of 1 s, starting with 0.1 mA, while being asked to rate how intense they perceived each stimulation on a VAS ranging from 0 (not intense at all) to 100 (maximum tolerable intensity/painful) using a joystick (Microsoft Corporation, Redmond, WA). The stimulation amplitude continued increasing until the participant first rated the sensation as 50 (mild pricking). Once 50 was reached, the amplitude was further increased by 0.1 mA, and if the participant rated the stimulation as higher than 50, it was then decreased by 0.1 mA until the rating went back to 50. This procedure was repeated until the participant rated the same amplitude as 50 on the VAS three times in a row or until the maximum amplitude of 5 mA was reached.

Following the amplitude calibration, the lights were turned off and the curtains were closed while participants were seated with their heads on a chin rest. A small ambient light was placed behind the participant to ensure that the pupils would not be fully dilated to avoid ceiling effects. For pupillometry, we used an EyeLink 1000 (SR Research Ltd., Kanata, ON, Canada) with a desktop mount. The eye-tracker camera was positioned ∼80 cm from the participant in front of the PC used for the experiment, while the screen was placed ∼95 cm from the participant. The eye-tracker was calibrated and validated using a 9-point horizontal-vertical procedure provided by EyeLink, asking the participant to follow a dot moving around the screen with their eyes. We performed the eye-tracker calibration and validation once after each amplitude calibration, and we re-validated it with a 1-point drift correction procedure before each block to adjust for slight movements of the participant.

Once the eye-tracker was fully calibrated, a first block consisting of 15 trials with the screen changing from a light gray (RGB #808080) to a black (RGB #000000) background every 15 s without stimulation was recorded. This luminance block was implemented as a control to check potential differences in general pupil dilation unrelated to vagal stimulation. The subsequent three blocks consisted of 15 taVNS pulses of 1s followed by an interstimulus interval of 25s. Each block took approximately 6 min, after which the participant was asked to rate how intense the stimulation was perceived on a scale from 0 (not intense at all) to 100 (maximum tolerable intensity). After three blocks of one condition (taVNS or sham) were completed, the participant could take a short break. Then the same procedure was repeated for the other condition starting with the calibration of the individual stimulation amplitudes. At the end of the session, participants were once again asked to rate their current mood and metabolic state on VAS scales (Figure 5).

#### taVNS device

Stimulation of the auricular branch of the vagus nerve was applied transcutaneously using the tVNS Technologies R device (Reichenschwand, Germany). Pulsed taVNS was delivered as 1s bursts of stimulation with a pulse width of 400μs, and a frequency of 20Hz. For the taVNS condition, the electrodes were placed at the cymba conchae of the right ear. For the sham condition, the electrodes were turned upside-down and placed on the right earlobe.^5^^,^^8^^,^^12^^,^^40^^,^^46^

### Quantification and statistical analysis

#### Power analysis

As this experiment was part of a larger study, we planned the sample size to accommodate a follow-up fMRI study using taVNS. For this fMRI study, our power (two-tailed test for paired means) analysis indicated a sample size of 40 participants to successfully detect a moderate *d*_*z*_ of 0.50 with a power of 0.80 and α = 0.05. To achieve this number, we recruited participants for the initial eye-tracking session until 40 taVNS responders (i.e., individuals with greater maximum dilation for taVNS vs. sham in the 5s post stimulation phase) completed the fMRI part of the study. This was achieved after testing 96 individuals (44 responders, 3 dropped out after the eye-tracking sessions). After removing incomplete sessions (*N* = 1 with data loss due to a malfunctioning of the eye-tracker) and data containing too many artifacts (*N* = 1), we included 94 participants in our final analyses. This sample size provides excellent power (1-*β* = 0.97) for a Cohen’s *d*_*z*_ of 0.34 (i.e., effect size estimated from our meta-analysis on pulsed taVNS^28^).

#### Pupil preprocessing

Pupil dilation for both the left and right eye was recorded with the EyeLink 1000 eye-tracker at 1000Hz. To maximize data quality and reduce movement artifacts, participants used a chin rest that was fixed to the table. The pupil signal was simultaneously sent out as an analog signal to the stimulation host PC (SR Research analog card model, SR Research, Kanata, ON, Canada), digitized at 1000 Hz, and recorded by MATLAB (MATLAB R2023b, The MathWorks). To exclude excessive noise, we applied a low-pass filter at 6Hz to the original time series and down sampled to 100 Hz. The preprocessing of the recorded signals was conducted using an in-house MATLAB pipeline adapted from D’Agostini and colleagues.^32^ First, we detected artifacts connected to blinks and merged consecutive blinks (with less than 0.25 s between blinks) into a single blink. We then padded the blink periods by 150 ms before and after each blink and linearly interpolated over the blink period. After initial interpolation, we detected and removed artifacts based on a temporal derivative threshold of 0.75 SD; the threshold was used to identify the start and endpoints of artifacts surrounding the invalid window. To achieve this, we extracted the absolute derivative value across the time series and defined as start/stop points those points followed by 60 ms in which the pupil measurement’s derivative value was below the 0.75 SD threshold. We set the limits for start and endpoints to 150 ms before and 250 ms after the invalid window. Finally, we interpolated the pupil signal between all invalid windows.

Second, we defined another derivative threshold of 3.5 SD and used it to identify remaining artifacts by repeating the first steps to identify potentially remaining invalid windows and, finally, interpolated between the windows. We separated the data into trials, with each trial starting with the beginning of stimulation and ending immediately before the beginning of the next stimulation. Each trial lasted 26s (1s of stimulation, 25s of interstimulus interval). We then obtained baseline pupil size from the second before the beginning of each trial (i.e., 1s before the start of the stimulation), and subtracted the baseline value corresponding to the trial from each value contained in the trial. Once we obtained baseline-corrected pupil size by subtracting the baseline and calculating the percentage change relative to the baseline values, we extracted our outcome variables. As main outcome variables, we defined maximum percentage pupil dilation as the peak value of baseline-corrected pupil dilation within the 5s post stimulation window.^36^^,^^53^ To reflect the response magnitude, we also calculated the area under the curve (AUC) using the *ROCR* package v1.0.11 in R^54^^,^^55^ for the pupil dilation trajectory between 0 and 5s. We further calculated the peak latency for each trial. We excluded samples with interpolated windows larger than 1s, as well as trials with more than 50% of missing or interpolated data (e.g., due to blinking), leading to the removal of 127 out of 8460 trials (1.50%).

#### Linear mixed-effects models

To analyze taVNS-induced changes in pupil dilation, we applied linear mixed-effects models using *lmerTest* v3.1.3 in R^51^, using the Satterthwaite method to determine the degrees of freedom and setting significance levels at α < 0.05. Mixed-effects analyses allow us to estimate fixed effects of stimulation and other design factors (e.g., characteristics common to the group), random effects (i.e., consistent individual deviations from the group average), and noise (i.e., non-reproducible variations caused by measurement error). Our model included fixed effects for stimulation condition (effect centered: sham = −0.5, taVNS = 0.5), block (linear term, coded as 0,1,2), stimulation amplitude (z-standardized), average rating differences (reflecting differences in sensation between conditions averaged across blocks) between taVNS and sham stimulation (taVNS - sham; centered and transformed to a range within −1 to 1 by dividing by 100), and condition order (effect centered: sham before taVNS = −0.5, taVNS before sham = 0.5), as well as interactions of stimulation condition with the other fixed effects. We decided to include rating differences to improve the estimation of the model (due to high correlations of estimated terms if absolute sensation ratings were used). This choice corresponds with the design as we matched sensations and accounted for potential residual variance, while amplitude differences arose from this procedure. However, since amplitude is associated with sensations, this cannot be fully disentangled with our design. At the participant level, the model contained random intercepts and slopes for stimulation condition and block:

maxpupildilation∼stimulation+block+ratingdifference+amplitude+order+stimulation∗amplitude+block∗stimulation+stimulation∗ratingdifference+order∗stimulation+(1+stimulation+block|ID)

We then applied the same model for 100 ms bins of the pupil dilation timeseries to explore whether our aggregate measures missed effects at specific times of the pupil response. We thus estimated separate mixed-effects models for pupil dilation binned into 100 ms intervals using the same fixed and random effects structure as in the main model. For each time bin, *t*-values and corresponding *p*-values were extracted for all fixed effects. To control for multiple comparisons across all time bins and model terms, we applied false discovery rate (FDR) correction (Benjamini–Hochberg method^56^) to the resulting *p*-values. Time points were considered significant if they survived FDR correction at α = 0.05.

To explore whether the stimulation amplitude or rated stimulation sensation was affected by stimulation order or differed in responders (vs. non-responders), we ran mixed-effects models with fixed effects for stimulation condition, responder status, or condition order, and their interaction. In addition, the models included random slopes for stimulation condition and a random intercept at the participant level:

meanamplitude∼stimulation+responder+stimulation∗responder+(1+stimulation|ID)

meanrating∼stimulation+responder+stimulation∗responder+(1+stimulation|ID)

meanamplitude∼stimulation+order+stimulation∗order+(1+stimulation|ID)

meanrating∼stimulation+order+stimulation∗order+(1+stimulation|ID)

The asterisks in the figures indicate significant differences with α < 0.05 and are calculated using linear mixed models with *lmerTest* v3.1.3 in R^51^ and the Satterthwaite method to determine the degrees of freedom.

To quantify the synchronization between left and right pupil dilation over time, we computed the correlation between the two pupil traces for binned time points of 10 ms across trials for each participant. To examine if taVNS affected the correlation of the pupil size of the left and right eye, we compared the correlation during stimulation (i.e., “early”, all 10 ms time bins from 1s before stimulation onset until stimulation end) with the correlation after stimulation (i.e., “late”, all 10 ms time bins from 1s to 5s after the end of stimulation). Correlation values were then Fisher z-transformed to normalize their distribution before statistical analysis. We then fit a linear mixed-effects model to the z-transformed correlation values, including fixed effects for stimulation condition (dummy-coded: sham (reference category) and taVNS), time segment (dummy-coded: early: baseline and stimulation period (reference condition), late: post-stimulation), and their interaction. The model also included random effects for both stimulation condition, time, and their interaction at the participant level:

Correlation(atanh−transformed)∼stimulation∗time+(1+stimulation∗time|ID).

Finally, to see whether responders and non-responders differed in the correlation between left and right pupil dilation, we added a fixed effect coding whether the participant was a responder to taVNS (dummy coded, non-responders as the reference category). In a further model, we included a fixed effect for rating differences (to reflect differences in sensation) to test a potential effect of sensation on the correlation between pupils.

#### Statistical threshold and software

All analyses were conducted with R (v4.2.1^54^) using ‘lmerTest’.^57^ Plots were made using the ‘ggplot2’ v3.4.4 and ‘sjPlot’ v2.8.16 packages.^58^^,^^59^ Estimated marginal means were calculated using the ‘emmeans’ package v1.10.5.^60^ We considered α < 0.05 as the significance threshold for all analyses.

### Additional resources

The study protocol was preregistered on clinicaltrials.gov (ID NCT06205108).
