# Supplementary material for: Effects of non-invasive vagus nerve stimulation on pupil dilation are dependent on sensory matching
Source: iScience. 2026 Jan 24;29(3):114795. doi: 10.1016/j.isci.2026.114795 (PMC12927277; doi:10.1016/j.isci.2026.114795)
Supplement: Document S1. Figures S1–S6 [file mmc1.pdf]

iScience, Volume *29*

Supplemental information

Effects of non-invasive vagus nerve stimulation on pupil dilation are dependent on sensory matching

Cecilia Vezzani, Rae-Marie Breakspear, Lilly Thurn, Ulrich Ettinger, Anne Kühnel, and Nils B. Kroemer

Supplemental Information

Figure S1. AUC model, and changes in pupil baseline between conditions, related to Figure 1.

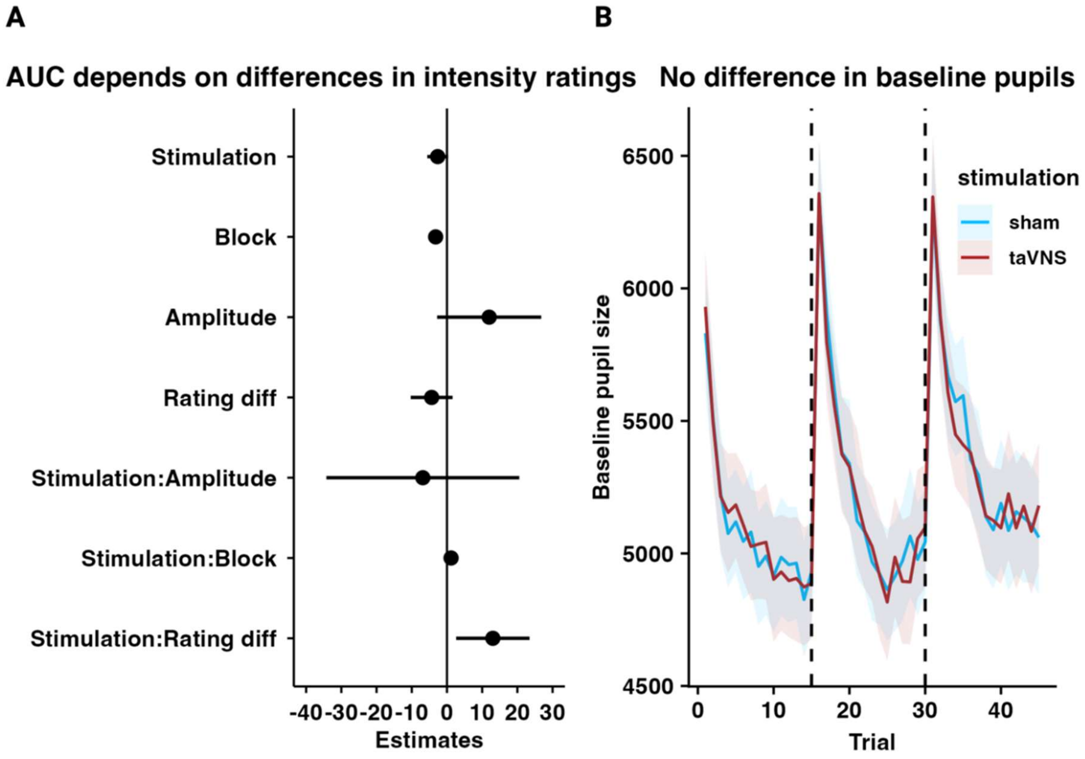

Figure S1. A) Mixed effects model for the area under the curve (AUC) for pupil dilation in the 5s post-stimulation, showing a negative effect for block (*b*(292.56) = -3.10, 95% CI [-4.14; -2.07], $p < .001$) and a positive interaction effect for stimulation and rating differences (*b*(93.91) = 13.76, 95% CI [3.30; 24.23], $p = .011$). B) Changes in baseline pupil size (used to subtract to the post-stimulation pupil size to calculate percentage pupil change), showing no difference between taVNS and sham (*b*(93) = 3.03, 95% CI [-61.54; 67.61], $p = .93$), but a sharp decrease over trials within each block (*b*(93) = -60.89, 95%CI [-70.19; -51.59], $p < .001$).

Figure S2. Decrease in pupil dilation over block and time-resolved analyses including order effects, related to Figure 2A.

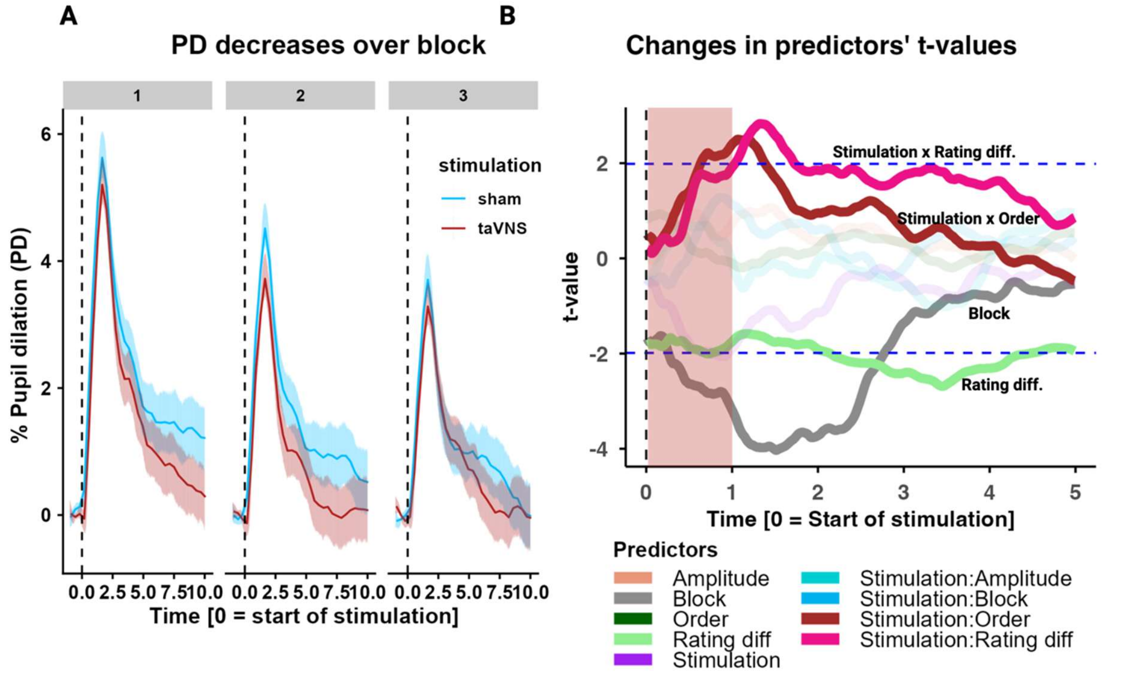

Figure S2: A) Decrease in pupil dilation across blocks for both taVNS and sham ($b(89.5) = -.97$, 95% CI [-1.23; -0.71], $p < .001$). B) Time-resolved analyses of taVNS-induced changes in pupil dilation show an early modulation by rating differences (Rating diff.) and order. Additional effects that cross the significance threshold are highlighted. The red overlay shows the time of stimulation. Data was analyzed in bins of 10ms.

Figure S3. Changes in sensation ratings over blocks and changes in pupil dilation depending on condition order, related to Figure 3 and Table 1.

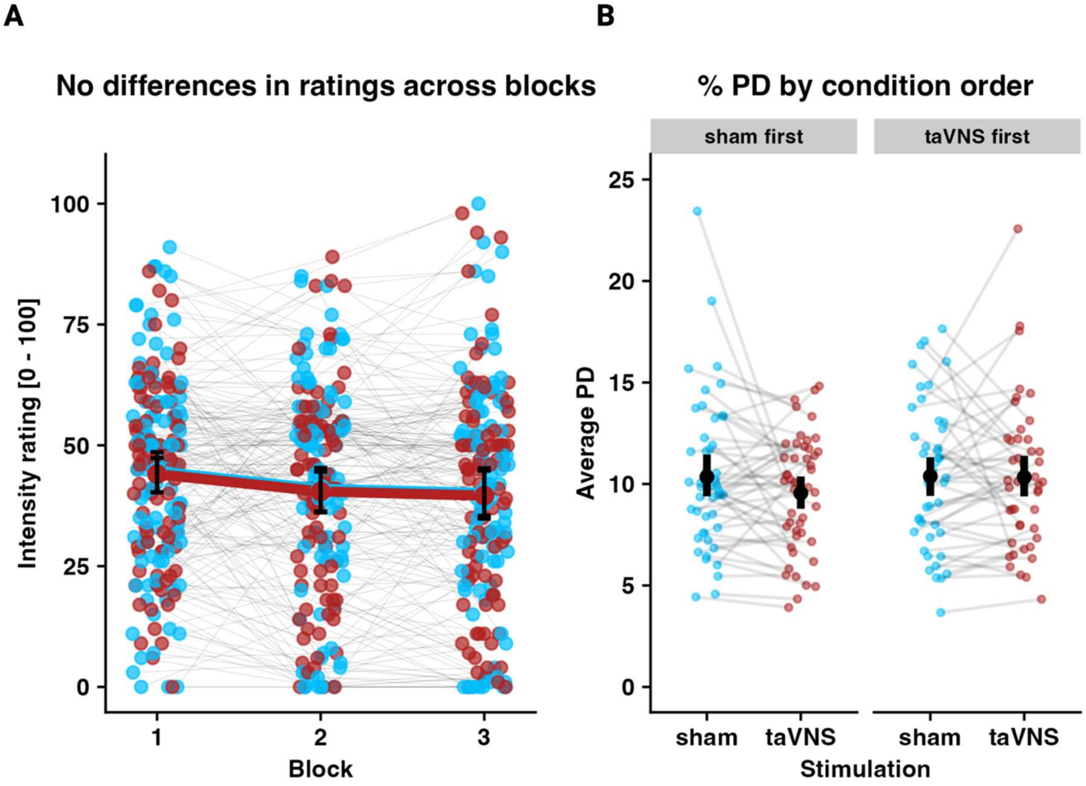

Figure S3: A) Decreases in sensation rating over blocks *b*(92) = -2.23, 95% CI [-3.72; -0.74], *p* = .003, but no significant interaction between blocks and stimulation (*b*(92) = 0.01, 95% CI [-2.63; 2.64], *p* = .99. B) Maximum pupil dilation (PD) does not change depending on condition order (*b*(93) = 0.18, 95% CI [-1.10; 1.46], *p* = .78), suggesting order did not affect pupil dilation.

Figure S4. Effect of order on amplitude and sensation ratings, related to Table 1.

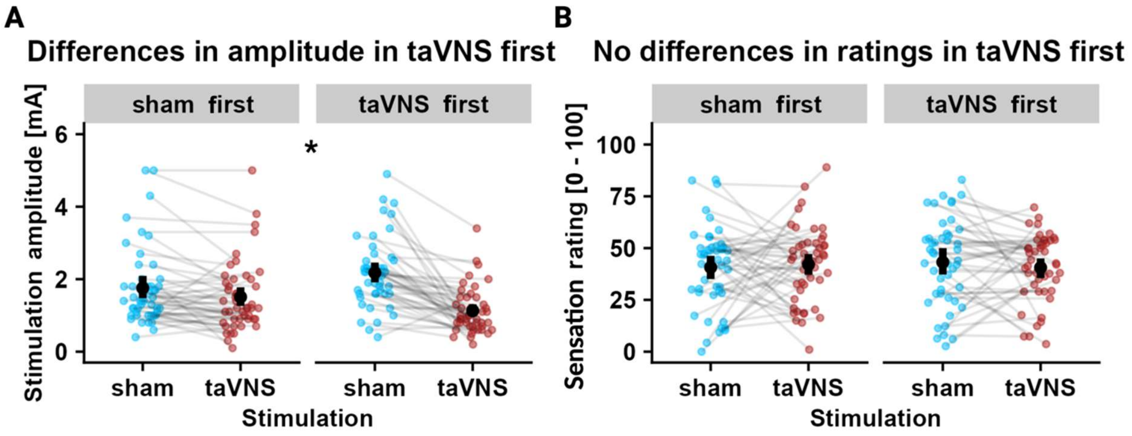

Figure S4: A) Lower amplitude for sham when sham was delivered first, *b*(131.7) = -0.42mA, 95% CI [-0.80; -0.05], *p* = .028), and higher difference in amplitude between taVNS and sham in the taVNS first condition, *b*(92) = -0.80mA, 95% CI [-1.12; -0.48], *p* < .001). B) No differences in sensation for taVNS vs. sham when separated by condition order, *b*(92) = -2.50, 95% CI [-10.75; 5.75], *p* = .55, (Order × Stimulation: *b*(92) = 4.04, 95% CI [-5.62; 13.70], *p* = .41). In all figures, error bars depict 95% bootstrapped CIs. *Significance levels are set at $\alpha < .05$ and are calculated through linear mixed models, using the Satterthwaite method to determine the degrees of freedom.

Figure S5. Changes in pupil dilation depending on start of testing time, related to Table 1.

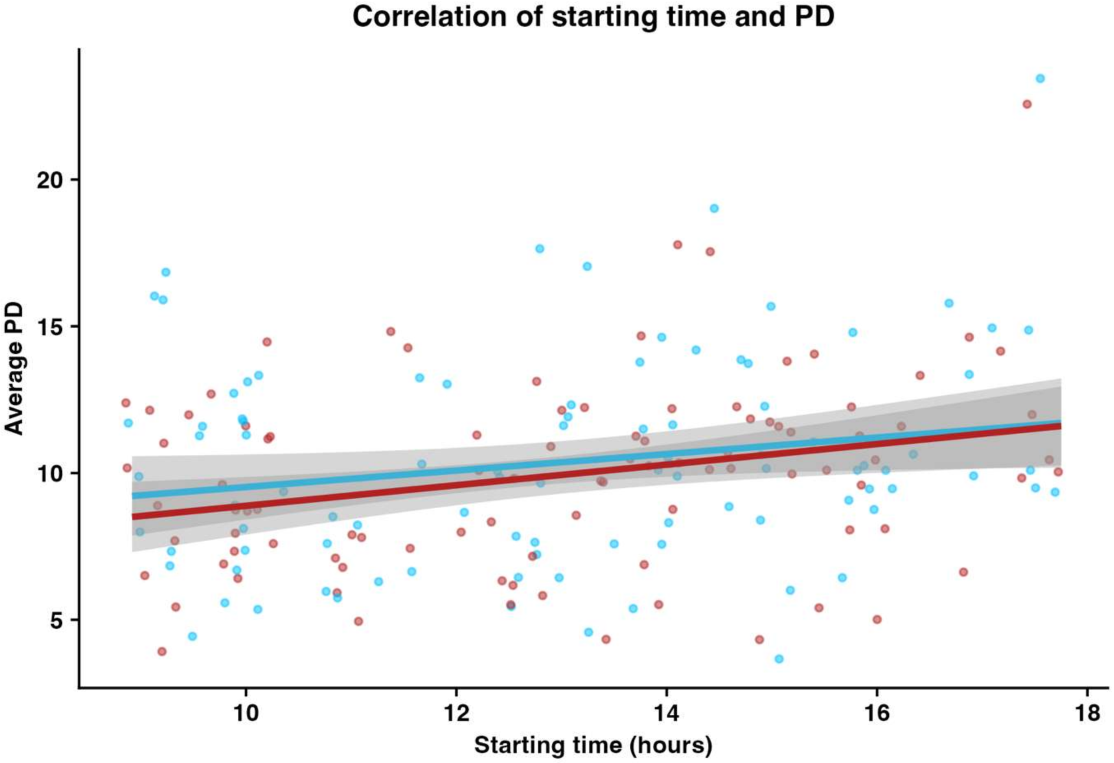

Figure S5: Positive correlation between average pupil dilation and time of the day showing a higher pupil dilation as the day progressed ($r(92) = 2.79$, $p = .006$), but no difference between taVNS and sham ($p > 0.05$).

Figure S6. Effect of block-wise ratings on pupil dilation, related to Figure 1.

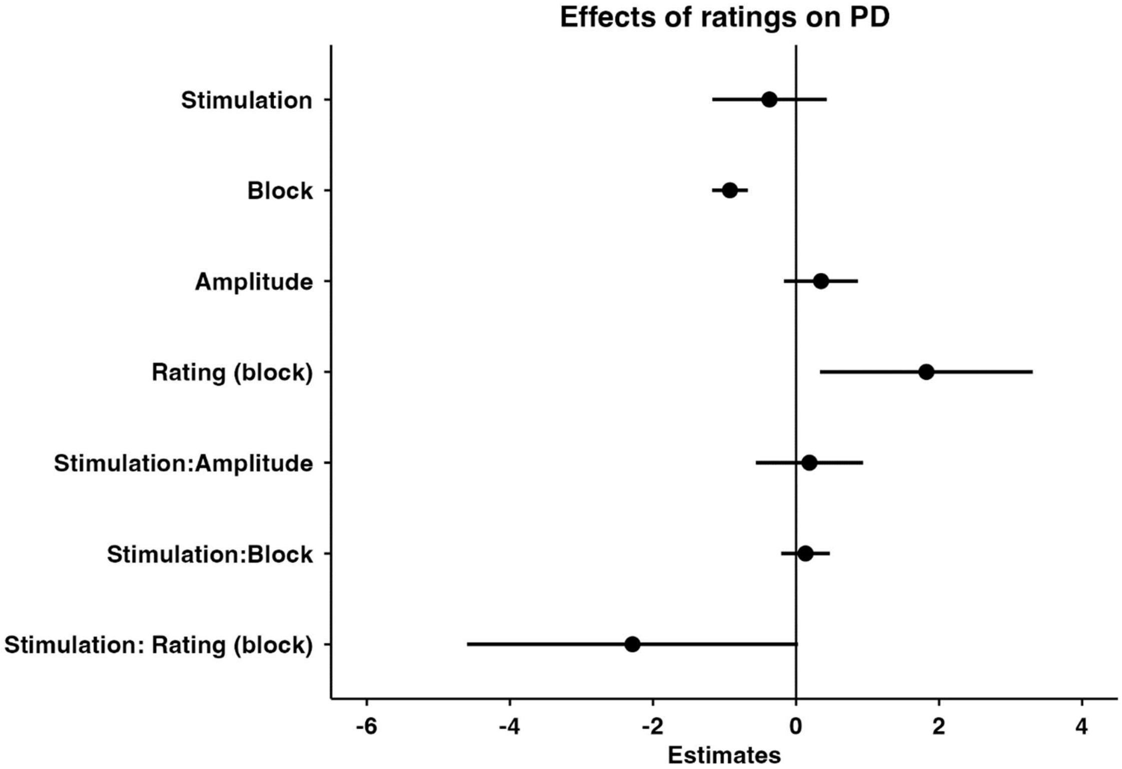

Figure S6: Mixed-effects model for the maximum pupil dilation including block-wise ratings in the model, showing a positive effect of rating, $b(45.45) = 1.82$, 95% CI [0.34; 3.31], $p = .016$ indicating that higher subjective ratings after each block were associated with greater pupil dilation. No significant interaction between stimulation and rating ($b(483) = -2.29$, 95% CI [-4.60; 0.03], $p = .053$;), in line with the outcome of the estimated marginal means suggesting that sham stimulation might produce more variable pupil dilation responses compared to taVNS.
